# The Influence of Myopia on the Foveal Avascular Zone and Density of Blood Vessels of the Macula—An OCTA Study

**DOI:** 10.3390/medicina59030452

**Published:** 2023-02-24

**Authors:** Maja L.J. Živković, Lazar Lazić, Marko Zlatanovic, Nevena Zlatanović, Mladen Brzaković, Mihailo Jovanović, Sava Barišić, Diana-Maria Darabus

**Affiliations:** 1 Ophthalmology Clinic, Clinical Center Niš, Bulevar dr Zorana Đinđića 48, 18000 Niš, Serbia; 2 Department of Ophthalmology, Faculty of Medicine, University of Niš, Bulevar dr Zorana Đinđića 81, 18000 Niš, Serbia; 3 Community Health Center Niš in Niš, Vojvode Tankosića 15, 18000 Niš, Serbia; 4 Special Hospital for Ophthalmology “Clinic Maja”, Vizantijski Bulevar 8, 18000 Nis, Serbia; 5 Department of Clinical and Experimental Surgery, Faculty of Medical Sciences, University of Kragujevac, Svetozara Markovića 69, 34000 Kragujevac, Serbia; 6 Eye Clinic, Clinical Center Vojvodina, Hajduk Veljkova 1-9, 21000 Novi Sad, Serbia; 7 Department of Ophthalmology, University of Medicine and Pharmacy “Victor Babes”, Piata Eftimie Murgu, No. 2, 300041 Timisoara, Romania

**Keywords:** OCTA, macula, vessel density, perfusion, myopia, foveal avascular zone (FAZ)

## Abstract

*Background and Objectives:* Myopia is the most common refractive eye anomaly with a prevalence that is constantly increasing. High myopia is associated with numerous complications that can lead to permanent vision loss. It is believed that the basis of these complications lies in changes in the microvasculature of the retina caused by an increase in the longitudinal axis of the eye. *Materials and Methods:* Optical coherence tomography angiography (OCTA) was used to analyze differences in macular zone vascular and perfusion density and foveal avascular zone (FAZ) parameters in myopic subjects. The following OCTA parameters were analyzed: the vessel and perfusion density of retinal blood vessels in the superficial plexus; the area, perimeter, and index of circularity of the foveal avascular zone (FAZ); and foveal and ganglion cell complex (GCC) thickness. *Results:* Subjects with low myopia did not show statistically significant differences compared to the control for any of the analyzed parameters. Groups with moderate and high myopia showed a significant decrease in vessel and perfusion density in the parafoveal and the entire 3 × 3 mm analyzed field. Foveal vessel and perfusion densities in the myopic groups were similar to those of the control regardless of the degree of myopia. The area and perimeter of the FAZ, as well as foveal and mean GCC thickness, did not differ significantly no matter the degree of myopia, while the index of circularity was lower in highly myopic subjects. The minimal thickness of the GCC was also lower in the high myopia group. *Conclusions:* High and moderate myopia led to a loss of blood vessels in the macular region. Perfusion and vascular densities were preserved in the foveal region and were not affected by different degrees of myopia. The FAZ was not significantly larger in myopic subjects, but its circularity was lower in subjects with high myopia.

## 1. Introduction

Myopia, or nearsightedness, is the most common eye refractive error and also the most widespread ophthalmic disorder in the world, with an average prevalence of about 25% in the adult population [1,2]. Myopia shows a worryingly rapid trend of incidence increase; it is estimated that 49.8% of the world’s population will suffer from it by 2050 [2]. The prevalence of axial myopia has profoundly increased over the last three decades globally in the younger generations, mostly related to the combination of increased urbanization, the pronounced intensification of education, and a marked reduction in time spent outdoors. Since the main risk factor for the development of pathologic complications of myopia in adulthood can be associated with axial elongation, it can be predicted that later in life a large number of presently young myopic individuals may develop pathology related to myopia [3,4].

High myopia, which is defined as the need for a spherical equivalent (SE) above −6 diopters (D), is a significant cause of permanent visual impairment. The main pathophysiological mechanism of high myopia is an increase in axial length (AL) which causes chorioretinal stretching and thinning [5,6]. The generally accepted principle is that for every increase in AL by 1 mm, SE increases by −3 D (various research results show that the range can vary from −3.33 to −1.24 D for 1 mm of growth) [7,8]. High myopia is associated with a number of complications such as: cataracts, chorioretinal atrophy, macular holes with or without retinal ablation, myopic foveoschisis, changes in the papilla of the optic nerve, posterior staphyloma, and others [9]. These complications are highly dependent on morphological changes in retinal blood vessels [10]; therefore, early monitoring of macular microvasculature alterations in young people with high myopia can represent a significant prophylactic measure and enable the early application of effective therapy [10]. Visual acuity depends on macula lutea. The configuration of the neuroretinal layer in the area of the macula must be within physiological limits without any pathological changes. As the receptors in the area of the fovea are fed by the surrounding capillary network from the retina and choroid, the submacular area of the choroid must be also preserved and orderly. This circular area in the center of the macula represents the FAZ. One of the main characteristics of axial myopia is that the length of the eye makes the retina and choroid become more stretched, which results in morphological changes in the FAZ and consequently reduces visual acuity.

Therefore, every examination of patients with minus diopters must include a wide-pupil examination of posterior parts of the eye. With this examination, irregularities and pathological changes which can lead to complications with the consequence of impaired vision can be observed. Depending on the clinical findings on the fundus, additional diagnostics such as eye ultrasound and OCT should be performed.

Optical coherence tomography (OCT) has made one of the biggest strides in the field of visualization methods in ophthalmology. Based on this platform, OCT angiography (OCTA) allows us to obtain images of blood vessels and blood flow at different levels of the retina and choroid without the use of contrast, with an amount of detail that is far above that of the old imaging techniques [11]. Although OCTA is very young method (having first entered commercial use in 2014), its potential application in the early detection of retinal vascular pathologies and elucidation of the pathogenesis of various ophthalmic diseases was quickly noticed and accepted with great enthusiasm. The principle of OCTA is based on the recording of fast, successive B-scans, which, due to the fact that the eye is stationary during the examination, give very similar images whose only differences are the changes in blood flow. By computer comparison of one B-scan with another—and based on the reflection and scattering of light on erythrocytes in blood vessels—a final picture of the vascular network can be obtained [11]. Early detection, accurate diagnosis, prognostication, and evaluation of treatment for myopia have been made easier with the application of imaging techniques, increasing their importance [12].

In current practice, the most frequently used tool for ophthalmic is optical coherence tomography (OCT), an emerging technology for performing high-resolution cross-sectional imaging. The pathological changes of myopic eyes only be investigated in enucleated eyes, histologically, before the implementation of OCT. Currently, OCT can be applied for imaging characteristic changes in ocular tissue due to myopia [13], from the anterior to posterior segment of the eye, including the anterior chamber, optic nerve, cornea, retina, vitreous, choroid, and sclera, which has greatly improved the understanding of myopia and pathologic myopia. Further, the development of OCT angiography (OCTA) has enabled the production of images of the blood flow of all the vascular layers with unprecedented resolution in a rapid, non-invasive manner, which can help detect vascular-related and myopia-related macular lesions such as myopic choroidal neovascularization (mCNV) and myopia-associated glaucoma-like optic neuropathy [14].

The aim of our study was to investigate differences in macular zone vascular and perfusion density as well as FAZ parameters in subjects with myopia using OCTA.

## 2. Methods and Materials

All participants were recruited from the Special Hospital for Ophthalmology “Clinic Maja”, Nis, Serbia, between February 2018 and March 2022. This case-control study was approved by the Ethics Committee of the Special Hospital for Ophthalmology “Clinic Maja” on 15 December 2017 with approval number 0/15-12/2017 and was carried out according to the tenets of the Helsinki Declaration. Written informed consent was obtained from all participants.

In this study, 136 eyes from 68 subjects with myopia and 132 eyes from 66 healthy volunteers were examined. Subjects with myopia, which was defined as SE ≤ −0.5 D, were divided into three groups: low grade myopic (25 subjects; SE ≥ −3.0 D); moderate grade myopic (21 subjects; SE range from −3.25 up to −6.0 D); and high myopic subjects without associated pathological ocular findings (22 subjects; SE ≤ −6.0 D). All participants were without any ocular pathologic findings detected on the anterior and posterior segment and with a best-corrected visual acuity (BCVA) of 20/20. Inclusion criteria were as follows: subjects older than 18 years with intraocular pressure lower than 21 mmHg and no pathological findings during eye examination. Exclusion criteria were: opalescent ocular media, history of prior intraocular surgery or injury, severe dry eye syndrome, glaucoma, or diabetes mellitus (which might affect the ocular circulation), medication usage within two weeks of measurements, and existence of any signs of pathological myopia at fundoscopic examination (chorioretinal atrophy, lacquer cracks, lattice degeneration, staphylomas, or paving-stone degeneration). Subjects with other retinal diseases such as: age-related macular degeneration, macular holes, epiretinal membrane, or extensive chorioretinal atrophy, as well as participants with an allergy to tropicamide, history of prior systemic diseases, and pregnancy, were also excluded. These criteria were established with the aim of excluding other eye diseases that may have affected the tested parameters, and so the presented results reflect only the changes caused by myopia.

**Figure 1:**
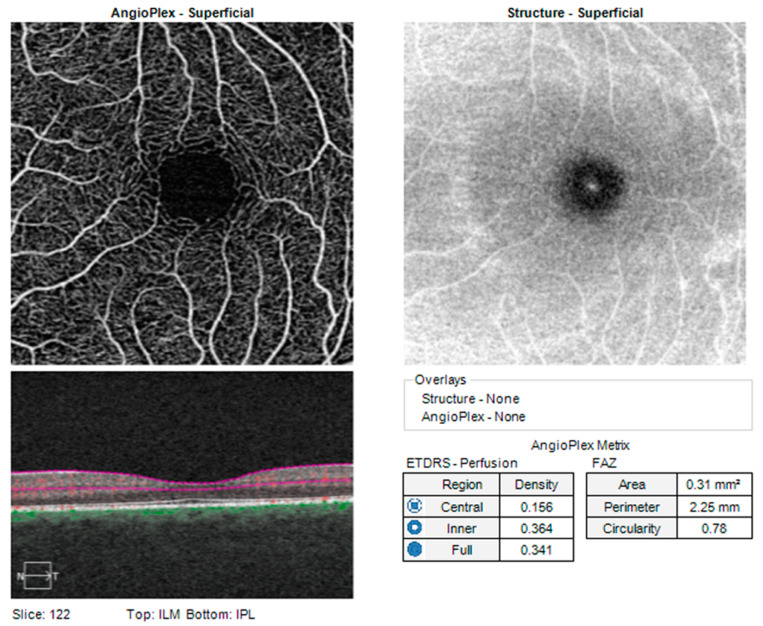
Standard report of a healthy patient obtained with a Cirrus HD-OCT device, Carl Zeiss Inc (ILM—Internal limiting membrane; IPL—Inner plexiform laser; FAZ—Foveal avascular zone; ETDRS—Early treatment diabetic retinopathy study).

A complete ocular examination was performed on all participants, including determination of AL by a WaveLight OB820 (Alcon, a Novartis Division, Fort Worth, TX, USA) optical biometer. The OCT protocol was conducted according to standardized operating procedures using a Cirrus HD-OCT device, HD-OCT—High Definition Optical Coherence Tomography, (model 5000, software version 10.0, Carl Zeiss Meditec, Inc., Dublin, CA, USA). While clinicians primarily utilize OCT-A to qualitatively assess retinal microvasculature, researchers in recent years have aimed to develop more quantitative approaches. These include the development of several vascular metrics that aim to quantify vascular features such as density and morphology. The Ganglion Cell Analysis algorithm of the aforementioned device was used to process and measure the thickness of the macular GCL + IPL layer. The OCT parameters that we analyzed were the average GCL + IPL thickness (GCC-average) and minimal GCL + IPL thickness (GCC-min). OCT angiography images were obtained with a 68 kHz Cirrus HD-OCT 5000-based Optical Micro Angiography (OMAG) prototype system. The analyzed parameters such as the perfusion density and vessel density of the retina and the area, perimeter, and circularity of the foveal avascular zone (FAZ) were automatically determined using the built-in software on the Zeiss AngioPlex OCTA device (Carl Zeiss Meditec, Inc., Dublin, CA, USA). The acquired images were checked for quality (signal strength of more than 6/10) as well as the absence of artifacts. As is shown in Figure 1, the AngioPlex software automatically divides the retina, according to an ETDRS map, into a central (1 × 1 mm) segment and inner (between 1 and 3 mm) segment and calculates the density and perfusion of the superficial capillary plexus blood vessels in them (we labeled these values as Vessel-central, Vessel-inner, Perfusion-central, and Perfusion-inner). The software can also calculate the vessel and perfusion density at the level of the entire analyzed 3 × 3 mm segment (labeled as Vessel-full and Perfusion-full). Vessel density is defined as the total length of perfused vasculature per unit area in a region of measurement, and the perfusion density is defined as the total area of perfused vasculature per unit area in a region of measurement.

Currently, the Zeiss AngioPlex OCTA allows access to 3 × 3, 6 × 6, and 8 × 8 mm scans. In this study, considering that it was very important to get accurate measurements of the FAZ, 3 × 3 mm scans were used exclusively. The circularity index was also calculated, which describes how similar a geometric object is to a perfect circle. A perfect circle has a circularity index of 1, while a thin thread-like object would have this index approaching 0. Equation (1) for determining circularity is as follows:

(1) $$\frac{4\pi A}{P^{2}}$$

where *A* is the area of the analyzed shape and *P* is its perimeter.

Descriptive data were presented as mean and standard deviation (SD). Categorical variables were expressed as frequency and percentages. To assess differences among groups, ANOVA with Bonferroni correction was used. All analytical procedures were performed using the Statistical Package for the Social Sciences (SPSS) for Windows, Version 22 (SPSS Inc.; Chicago, IL, USA). A value of *p* ≤ 0.05 was considered as statistically significant.

## 3. Results

This study included a total of 136 eyes of 68 subjects who had myopia. Table 1 presents demographic data for the participants included in this study. Of the 68 subjects, 38 (55.9%) were female and 30 (44.1%) were male. The mean age was 32.85 ± 8.56 years (the mean age of the women was 34.38 ± 9.23 years and for the men was 30.40 ± 6.90 years). Subjects were divided into three subgroups based on SE values: group 1, *n* = 25 (−3 < SE ≤ −0.5); group 2, *n* = 21 (−6 < SE ≤ −3); and group 3, *n* = 22 (SE ≤ −6).

The control group included 66 subjects, i.e., 132 eyes. Out of the total number of subjects, 36 (54.54%) were female and 30 (45.46%) were male. The mean age was 34.43 ± 9.68 years (the mean age of the women was 32.33 ± 3.64 years and for the men was 37.22 ± 13.91 years). Subjects did not differ significantly according to age (*p* = 0.133) or sex (χ^2^ = 2.63, df = 3, *p* = 0.452). The difference between the involvement of the right or left eye was also insignificant (χ^2^ = 2.27, df = 3, *p* = 0.518). Table 2 presents the results of statistical analysis for the measured OCTA parameters.

As expected, the subjects differed in their AL values, both between the examined groups as well as during comparison with the control group (F (3.90) = 51.36, *p* < 0.001). Central subfield thickness (CST, also known as foveal thickness) among the groups was not significantly different (F(3.86) = 0.60, *p* = 0.618).

Vessel-central and Perfusion-central values did not differ significantly in any group of subjects compared to the control (F(3.88) = 1.35, *p* = 0.265 and F(3.88) = 1.16, *p* = 0.331, respectively). Values of Vessel-inner and Perfusion-inner as well as Vessel-full differed significantly in the group of subjects with moderate and high myopia compared to the control (*p =* 0.039, *p =* 0.036, *p =* 0.043 for moderate and *p =* 0.001, *p* = 0.001, *p* = 0.001 for high myopia, respectively). Perfusion-full was significantly lower in the group of subjects with high myopia (*p =* 0.003), while in the group with moderate myopia it was at the limit of statistical significance (*p* = 0.052).

The area and perimeter of the FAZ did not differ significantly between subjects regardless of the severity of myopia (F(3.83) = 0.94, *p* = 0.425 for area and F(3.83) = 0.29, *p* = 0.830 for perimeter). The index of circularity was reduced only in the group of subjects with high myopia compared to the control (*p =* 0.006 for comparison).

Participants did not differ significantly in terms of the GCC-average value (F(3.83) = 1.18, *p* = 0.321), while GCC-min was significantly reduced only in subjects with high myopia (F(3.83) = 5.33, *p =* 0.002 for ANOVA; *p =* 0.001 for comparison of high myopia vs. control).

## 4. Discussion

OCT angiography as a new imaging technique provides significantly more information about the morphology of the microvasculature with a better possibility of quantifying vascular alterations in retinal diseases. In this study, we used OCTA to analyze and quantify changes in vessel density as well as the reduction of flow in the superficial retinal plexus in myopic eyes compared to a healthy control. We also analyzed parameters of the FAZ: the area, perimeter, and its index of circularity as well as the thickness of the GCC and thickness of the fovea.

The results of our research show a statistically significant decrease in the density of blood vessels of the superficial vascular plexus in the groups of subjects with moderate and high myopia at the level of the inner analyzed segment (i.e., Vessel-inner; parafoveal region) as well as in the entire examined 3 × 3 mm field (i.e., Vessel-full) compared to the control. A similar trend was seen in the density of the perfused vasculature (i.e., “Perfusion” parameter), with the exception of comparison of the moderately myopic group’s Perfusion-full parameter with that of the control, which was at the limit of statistical significance (*p* = 0.052). There was no difference when comparing these parameters between different myopia. These results are in agreement with previous studies that reached similar results [5,10,15,16,17]. Shimada et al. [18] and Shi et al. [10] considered the increase of the eyeball’s AL as a possible explanation for this finding. Moreover, retinal thinning caused by mechanical stretching leads to retinal atrophy and reduced oxygen consumption. Since the number of blood vessels directly depends on the need for oxygen, along with the fact that retinal blood vessels lack autonomous innervation as a regulatory mechanism [19], this results in a decrease in vascular density. Furthermore, the possibility of easier diffusion of oxygen from the choroid (due to reduced thickness of the retina), which causes secondary loss of blood vessels, cannot be ruled out. Studies that have not obtained a decrease in the density of blood vessels in the parafoveal region may have achieved such results due to a different methodology or because of the small age span of the examined groups [20,21]. Choroid is rich in a large number of blood vessels, and the changes in its structure and function play a certain role in the development of myopia [22]. According to published results, choroidal thickness (ChT) is correlated with refractive diopters to a certain extent, and the higher the degree of myopia, the thinner the ChT [23]. Changes in the ocular refractive state can lead to rapid changes in ChT. One study with children found that hyperopic defocus caused choroidal thinning, while myopic defocus induced relative choroidal thickening [24].

We also failed to find a decrease in blood vessel and perfusion density in the central examined region (i.e., Vessel-central, foveal region), regardless of the severity of the myopia, which is consistent with previous studies in adults and children [15,21]. According to Wang et al. [15], this may be because the largest part of the foveal zone is represented by the FAZ, which is characterized by an active metabolism that maintains blood flow in a stable state which is not affected by the elongation of the eyeball.

FAZ is a capillary-free area inside the macular region, surrounded by interconnected capillary beds, which is the most sensitive area of vision; it mainly reflects the microcirculation status in the macular area. FAZ size may be related to gender, age, retinal thickness, AL, macular blood flow, segmentation method, RNFL thickness, etc. [25]. Shahlaee et al. found that the FAZ area of the superficial layer in normal adults was 0.27 ± 0.101 mm^2^, and that of the deep layer was 0.34 ± 0.116 mm^2^, as measured by OCTA [26]. The FAZ area of high myopic eyes was larger than that of emmetropia [27]. The area of the FAZ as well as its perimeter did not show statistically significant differences between the examined groups and the control. This is consistent with some studies [5,15], while in others the FAZ was larger than in the control group [17,28,29]. The reason for this finding could be the fact that the FAZ varies significantly among healthy people [30], and that no correlation was observed between AL and the FAZ [31]. However, as post hoc analysis showed, the index of circularity was significantly reduced in the group of highly myopic subjects compared to the control, suggesting a greater deviation of the contours of the FAZ from a regular circle. As far as we know, in addition to ours, there is only one other study that examined the index of circularity among healthy highly myopic subjects without added comorbidities [32]. The results of the aforementioned study, similar to ours, failed to show a significant decrease in the index of circularity when comparing this parameter among the high myopia group with the non-high myopia group, but unlike ours, this study lacked a control group, so a comparison between high myopia subjects and healthy volunteers could not be made. The results of our research suggest that circularity index and parafoveal vessel density might be a better parameter for monitoring macular vascular loss than the size and perimeter of the FAZ, which are subject to significant interpersonal variation. Future research will demonstrate the possible applicability of the circularity index in the follow-up of highly myopic subjects.

There was no statistically significant difference of foveal thickness in the examined groups compared to the control, which is in accordance with already published works [33]. Some studies have come to different results where the fovea was thicker in high myopia [34]. Possible reasons for this might be secondary elevation of the fovea caused by vitreoretinal traction [21] or increased permeability of the retinal pigment epithelium in the foveal region [35].

Previous studies have shown that retinal thickness in the macular area of myopic eyes is thinner than that of emmetropia due to the elongation of AL in myopic eyes [36]. However, the retina in the parafoveal and the peripheral macular areas is subjected to a greater tensile force of the sclera; thus, the retina in the parafoveal and the peripheral macular areas is more prone to thinning than that in the fovea [37]. Milani et al. reported that the superficial vessel density in the macular area of adults with high myopia was positively correlated with retinal thickness [31]. Wu et al. found that the retina and choroid in adults with high myopia were thinner, and there was a negative correlation between the thickness of the outer retina and the density of deep retinal vessels, suggesting that the deep retinal vessels might have a compensatory effect on the hypoxic environment of high myopia [38].

We also found that the average thickness of the GCC was not significantly reduced compared to the control regardless of the severity of myopia. The value of the minimal GCC thickness was lower in all three examined groups, but significantly so only when comparing the high myopia group to the control (*p =* 0.025). The majority of published studies have shown a statistically significant reduction in GCC thickness in high myopia, as well as a negative correlation between the thickness of the GCC and AL or a positive correlation between the thickness of the GCC and SE [39]. GCC thinning can be explained by the elongation of the eyeball and the consequent increase in AL, which contributes to the reduction of the thickness of the entire retina including the GCC. However, there are studies, such as ours, that failed to show the existence of a correlation between the thickness of the GCC and the degree of myopia [38]. Perhaps the reasons for these findings were the small examined group as well as other differences in methodology. The influence of aging on GCC thickness and macular thickness in general has been proven in some studies [40], but refuted in others [41]. In our research paper, the control and examined groups did not differ from each other in terms of age, so the effect of aging as a contributing factor on GCC thickness can be disregarded.

It is important to point out that Corvi et al. [42], after conducting a safety analysis, came to the conclusion that the variability between the values of the density of blood vessels of the superficial and deep plexus as well as the FAZ obtained on different OCTA devices is large, which makes the mutual comparison of values between devices of different manufacturers practically impossible. This highlights the importance of individual databases for each device model.

The presented study has several limitations: it is an observational, single-center, cross-sectional study, and the sample sizes are small, mono-racial, and have a narrow age range. Errors could also occur due to ocular magnification as a consequence of increased refraction, especially in high myopia [25,43].

In summary, vascular and perfusion densities in the parafoveal region as well as at the level of the entire examined 3 × 3 mm field were significantly reduced in moderately and highly myopic subjects compared to the controls. In the foveal region, the densities of blood vessels and perfusion were unchanged regardless of the degree of myopia. The area and perimeter of the FAZ did not differ significantly in subjects regardless of the degree of myopia, while the index of circularity was lower only in highly myopic subjects. Foveal thickness as well as the mean value of GCC thickness did not differ in the subjects regardless of the degree of myopia, while the minimal value of GCC thickness was only lower in highly myopic subjects. OCTA as a simple, non-invasive, practical technique could in the future offer early prevention of myopic retinopathy by providing an insight into the vascular changes of the retina.

## Figures and Tables

**Table 1:** Demographic data for study participants.

Demographic Data	Low Myopia Group (*n*_1_ = 25)	Moderate Myopia Group (*n*_2_ = 21)	High Myopia Group (*n*_3_ = 22)	Control Group (*n* = 66)	*p*
Age ($\bar{x}$ ± SD)	34.95 ± 9.20	32.35 ± 6.45	38.60 ± 6.87	34.43 ± 9.68	0.133
Sex, *n* (%)					
Male	9 (36.0)	11 (52.4)	6 (27.3)	28 (42.4)	0.452
Female	16 (64.0)	10 (47.6)	16 (72.7)	38 (57.6)	
BMI, ($\bar{x}$ ± SD)	24.25 ± 5.62	24.14 ± 2.99	24.12 ± 4.24	23.53 ± 4.04	0.914

Legend: BMI—body mass index.

**Table 2:** Results of statistical analysis for measured OCTA parameters.

	Low Myopia Group (*n*_1_ = 25)	Moderate Myopia Group (*n*_2_ = 21)	High Myopia Group (*n*_3_ = 22)	Control Group (*n* = 66)	*p* Value	Post Hoc
AL, mm	24.20 ± 0.47	25.19 ± 1.09	26.95 ± 2.17	22.37 ± 4.33	*p* < 0.001 *^a^	C < L < M < H
Foveal thickness, μm	260.45 ± 24.86	265.00 ± 26.46	258.62 ± 32.16	256.02 ± 16.97	*p* = 0.618	/
Vessel, mm^−1^						
central	11.75 ± 3.68	10.34 ± 3.79	10.04 ± 3.37	11.59 ± 2.81	0.265	/
inner	21.13 ± 2.22	20.61 ± 1.47	19.74 ± 3.06	22.13 ± 1.48	0.001 *^a^	M < C, H < C
full	20.06 ± 2.20	19.45 ± 1.54	18.62 ± 3.02	20.96 ± 1.50	0.001*^a^	M < C, H < C
Perfusion						
central	0.20 ± 0.06	0.18 ± 0.07	0.17 ± 0.06	0.20 ± 0.05	0.331	/
inner	0.38 ± 0.03	0.37 ± 0.03	0.36 ± 0.05	0.40 ± 0.02	0.001 *^a^	M < C, H < C
full	0.36 ± 0.04	0.35 ± 0.03	0.37 ± 0.05	0.37 ± 0.03	0.002 *^a^	H < C
Foveal avascular zone						
area	0.24 ± 0.11	0.26 ± 0.15	0.21 ± 0.08	0.26 ± 0.08	0.425	/
perimeter	2.06 ± 0.57	2.18 ± 0.74	2.05 ± 0.44	2.15 ± 0.37	0.830	/
index of circularity	0.68 ± 0.07	0.64 ± 0.13	0.61 ± 0.10	0.70 ± 0.07	0.004 *^a^	H < C
GCC thickness, μm						
average	81.05 ± 4.49	82.92 ± 7.80	83.25 ± 9.58	84.19 ± 4.99	0.321	/
minimal	77.15 ± 6.43	75.85 ± 5.94	70.25 ± 18.62	80.79 ± 4.35	0.002 *^a^	H < C

Legend: AL—axial length, GCC—ganglion cell complex, C—control group, L—low myopia group, M—moderate myopia group, H—high myopia group. * Statistically significant difference. ^a^ ANOVA.

## Data Availability

The authors confirm that the data supporting the findings of this study are available within the article.
